# Multi-bacillary leprosy under the Chinese leprosy elimination program^⋆^

**DOI:** 10.1016/j.abd.2021.08.012

**Published:** 2022-08-26

**Authors:** Ge Li, Chao Li, Qingping Zhang, Hong Zhang, Ping Chen, Zhaoxing Lin

**Affiliations:** a Shaanxi Provincial Institute for Endemic Disease Control, Xi’an, China; b School of Public Health, Xi’an Jiaotong University Health Science Center, Xi’an, China; c Department of Orthopedics, Xijing Hospital, The Fourth Military Medical University, Xi’an, China

Dear Editor,

Leprosy, caused by *Mycobacterium leprae*, is a chronic infectious disease, which may cause permanent damage to peripheral nerves and deformity.^1^ According to the World Health Organization (WHO), leprosy cases are classified^2^ in PB (paucibacillary) or MB (multibacillary) leprosy based on the number of skin lesions: PB leprosy (2‒5 skin lesions), and MB leprosy (more than 5 skin lesions). MB leprosy is mainly caused by the unresponsiveness of cellular immunity against leprosy bacilli,^3^ and is characterized by high infectivity and functional disability rate. Leprosy disability severely affects the life quality of leprosy patients and may induce psychological problems. However, despite effective measures were extensively implemented, the number of new cases worldwide has remained at almost 250,000 each year. Although leprosy is generally in a low endemic state in northwest China,^4^ the proportion of MB cases and the rate of disability are still at a high level.

To analyze the sociodemographic and clinical factors associated with MB leprosy in elimination planning areas in Northwest China, three specialized hospitals were included. The medical records of leprosy in province in northwest China from 2004 to 2020 were collected from the Leprosy Management Information System (LEPMIS).

This is an observational and retrospective study, involving 305 cases of MB and PB leprosy cases, collected in the LEMPIS from 2004 to 2020. The variables included gender, nationality, occupation, and others. The statistical significance level was p < 0.05. Logistic regression analysis was used to adjust the confounding variables to determine the independent risk factors of MB leprosy cases.

Significant differences in sex and some other variables (p < 0.05) were shown comparing MB and PB cases, as shown in Table 1. Of the MB leprosy cases, 21.32% cases were documented as having less than 5 skin lesions, as shown in Table 2.

**Table 1:** Relationship between sociodemographic and behavioral factors and leprosy types.

	MB leprosy (n = 272)	PB leprosy (n = 33)	p-value	OR	95% CI
**Sociodemographic factors**					
**Sex**					
Male	184 (67.65)	28 (84.85)	0.043	0.373	(0.139‒1.000)
Female	88 (32.35)	5 (15.15)			
**Ethnics**					
Han	268 (98.53)	33 (100.00)	0.483	0.89	(0.856‒0.926)
Others	4 (1.47)	0 (0.00)			
**Profession**					
Farmer	24 (8.82)	3 (9.09)	0.959	0.968	(0.275‒3.407)
Others	248 (91.18)	30 (90.91)			
**Education level, y**					
More than 9	6 (2.21)	0 (0.00)	0.002	3.145	(1.504‒6.573)
Between 1 and 9	184 (67.65)	14 (42.42)			
Less than 1	82 (30.15)	19 (57.58)			
**Marital status**					
Unmarried	69 (25.37)	20 (60.61)	<0.001	0.221	(0.104‒0.468)
Married	203 (74.63)	13 (39.39)			
**Living history**					
Local	267 (98.16)	30 (90.91)	0.014	5.34	(1.215‒23.465)
Nonlocal	5 (1.84)	3 (9.09)			
**Age, y**					
Older than 60	40 (14.71)	9 (27.27)	0.063	0.460	(0.199‒1.061)
Between 20 and 60	224 (82.35)	23 (69.70)			
Younger than 20	8 (2.94)	1 (3.03)			
**Behavioral factors**					
Mode of diagnosis					
Active detection	53 (19.49)	15 (45.45)	0.001	0.290	(0.137‒0.614)
Passive detection	219 (80.51)	18 (54.55)

**Table 2:** Relationship between clinical factors and leprosy types.

	MB leprosy (n = 272)	PB leprosy (n = 33)	p-value	OR	95% CI
**Skin lesion**					
None	8 (2.94)	5 (15.15)	< 0.001	0.170	(0.052‒0.554)
1 lesion	6 (2.21)	7 (21.21)			
2‒5 lesions	44 (16.18)	15 (45.45)			
>5 lesions	214 (78.68)	6 (18.18)			
**Leprosy reaction**					
No reaction	233 (85.66)	30 (90.91)	0.3	0.597	(0.174‒2.053)
I reaction	15 (5.51)	3 (9.09)			
II reaction	20 (7.35)	0 (0.00)			
Mixed reaction	4 (1.47)	0 (0.00)			
**Skin smear result**					
Positive	177 (65.07)	4 (12.12)	< 0.001	13.508	(4.612‒39.566)
Negative	95 (34.93)	29 (87.88)			
**Nerve damage**					
None	53 (19.49)	5 (15.15)	0.003	1.355	(0.500‒3.678)
1 nerve	31 (11.40)	11 (33.33)			
≥ 2 nerves	188 (69.12)	17 (51.52)			
**Disability**					
None	61 (22.43)	11 (33.33)	0.007	0.578	(0.266‒1.259)
Grade I	93 (34.19)	3 (9.09)			
Grade II	103 (37.87)	19 (57.58)			
Not clear	15 (5.51)	0 (0.00)			

These results showed high endemic characteristics of leprosy, suggesting that the delay on leprosy diagnosis^5^ still exists in northwest China, which led to more serious consequences and disabilities. Leprosy showed a low prevalence trend at this stage, and male patients still accounted for a relatively high proportion of the newly diagnosed cases in each year, which may be related to different genetic susceptibility in different genders. Meanwhile, females got more skin consultations than males, and males may be more easily exposed to leprosy bacilli related to behavioral^6^ and cultural factors, which may partly explain the dominant position of male cases. The susceptibility of the elderly to MB leprosy may be related to the prolonged incubation period of leprosy bacilli, resulting to delayed response. In addition, the aging of the immune system in the elderly^7^ was an aggravating factor for infection control. People with higher education^8^ are more inclined to seek medical services to avoid delaying diagnosis and treatment. Data showed that people with a marriage history are the advantaged group of MB leprosy, which may indicate that close contact in the home was related to exposure to leprosy bacilli, but the we cannot rule out the importance of social contact in disease transmission.

Passive detection was a protective factor for MB leprosy, so it is necessary to increase the publicity and education of leprosy prevention and control knowledge in low-prevalence areas, and to improve the awareness^9^ rate of the masses and self-care awareness. More than 5 lesions were associated with MB leprosy, which indicated that a high concentration of leprosy bacilli infection can lead to more tissue destruction, more skin damage, and worse deformation. MB cases have a higher probability of grade I or II disability after model adjustment. The incidence rate of Class II disability in the present sample is far higher than the global average of 6% reported by the WHO^10^ in 2016, which indicated that there is a delay in diagnosis or misdiagnosis in these patients.

This study was based on second-hand data obtained from the LEPMIS, so it has some limitations, such as inconsistent information, prevalence bias and the defect of cross-sectional design. Future longitudinal studies or geographical distribution are desirable to clarify factors related to leprosy.

In conclusion, MB patients were the main infectious source of leprosy, so early detection and treatment can effectively block the transmission of leprosy in the infectious source control link, which is of great significance to reduce the probability of leprosy in patients with disability. This study has shown the epidemic characteristics and regional characteristics of MB leprosy in northwest China, which may be helpful in effectively preventing and controlling leprosy.

## Financial support

None declared.

## Authors’ contributions

Ge Li: Co-wrote the article; the corresponding author, reviewed the data and results of this article; contributed to interpretation of the data, commented on the manuscript, revised the manuscript, and approved the final version for publication.

Chao Li: Co-wrote the article; contributed to interpretation of the data, commented on the manuscript, revised the manuscript, and approved the final version for publication.

Qingping Zhang: The corresponding author, reviewed the data and results of this article; contributed to the interpretation of the data, commented on the manuscript, revised the manuscript, and approved the final version for publication.

Hong Zhang: Contributed to the collection and acquisition of the article data; contributed to interpretation of the data, commented on the manuscript, revised the manuscript, and approved the final version for publication.

Ping Chen: Contributed to the collection and acquisition of the article data; contributed to interpretation of the data, commented on the manuscript, revised the manuscript, and approved the final version for publication.

Zhaoxing Lin: Gave guidance on research methods and case review; contributed to interpretation of the data, commented on the manuscript, revised the manuscript, and approved the final version for publication.

## Conflicts of interest

None declared.
